# Supplementary material for: Prevalence and risk factors of hypertension in Mauritius: A cross-sectional study
Source: PLOS Glob Public Health. 2024 Dec 12;4(12):e0003495. doi: 10.1371/journal.pgph.0003495 (PMC11637371; doi:10.1371/journal.pgph.0003495)
Supplement: Attachment — Submitted filename: Response to reviewers 2024.06.22.docx [file pgph.0003495.s001.docx]

5. Review Comments to the Author

Please use the space provided to explain your answers to the questions above. You may also include additional comments for the author, including concerns about dual publication, research ethics, or publication ethics. (Please upload your review as an attachment if it exceeds 20,000 characters)

Dear Editor,

We are deeply grateful to you and the reviewers for dedicating your valuable time to evaluate our manuscript and for offering insightful feedback. All comments were instrumental in enhancing the quality of our paper. We have addressed each suggestion and believe that the revised manuscript reflects these improvements. We remain open to any additional feedback you might have.

Below, you will find our detailed responses to each point raised. All changes in the manuscript have been include using track changes. Text from the manuscript is presented in cursive and indented. References to modifications in the text pertain to the "Revised manuscript (marked-up copy)."

Thank you for your consideration.

Best regards,

Osvaldo Fonseca Rodríguez on behalf of the authors

Reviewer #1: Review comments – PLOS Global Public Health
The paper is interesting and could contribute to bridging literature gap on hypertension prevalence and risk factors in Mauritius. However, the manuscript does not merit publication at it’s current state.

Abstract
Lines 42-43: A cross-sectional national representative data obtained from the 2021 Mauritius non-communicable diseases survey was used for this study. “This statement does not improve readability. I suggest the authors revise the statement to “A cross-sectional study was conducted using nationally representative data from the 2021 Mauritius non-communicable diseases survey”.

--- Thank you for helping us to improve the readability of the manuscript. The sentence was replaced in the revised version as according to your suggestion.

Introduction

Lines 73 – 78: The lines 73 – 78 talks about the prevalence and risk factors associated with hypertension in Mauritius. If you are aware of the risk factors of hypertension in Mauritius, what is the motivation for conducting this study?

--- The motivation for conducting the study on hypertension in Mauritius lies in the need for more comprehensive information about risk factors. Lines 73-78 provide information about the burden of hypertension in the country but not on the specific risk factors.

Lines 88 – 94: Lines 88 – 94 emphasise on the justification of the study. The authors should strengthen their study justification as it is weak in its current state. Why are they conducting the study? Is it to bridge literature gap in Mauritius?

--- As previously mentioned, there is lack of literature about the factors associated to hypertension in the country. In order to strengthen the justification to conduct the current study, we have now added the following text to the introduction of the manuscript (please, see page 4, lines 84-91):

*“The stable prevalence of hypertension despite existing health policies underscores a critical gap in our understanding of the current risk factors contributing to hypertension in Mauritius. Given the evolving sociodemographic and lifestyle patterns, it is imperative to update and expand the existing knowledge on the prevalence and determinants of hypertension. This study aimed to addressing this gap by investigating the prevalence of hypertension and associated factors in Mauritius in 2021. By identifying and analyzing these factors, our research will offer crucial insights necessary for tailoring public health strategies and interventions, and thus address health inequalities in the country* *[17].”*

Materials and Methods
There is no sub-section called “study design” in the methods section which is major weakness of the study. The authors should consider having a separate paragraph to present on study design.

--- We gave a slightly different structure to the methods by including study design section. In the revised version of the manuscript, we explicitly mention the type of study.

Study setting
The information on study setting is too scanty. The authors can add on.

--- The section “study setting” was extended in the revised version of the manuscript.

The following text was added to page 5, lines 119-121:

*“The Mauritian Ministry of Health and Wellness, in partnership with international research institutions, has conducted a total of seven population surveys on non-communicable disease risk factors since 1987.”*

Dependent variables
Lines 111 – 118: Trained personnel followed the standardized protocol to measure participants' blood pressure during the survey. Before measurement, participants rested for five minutes and the Omron M7 automatic blood pressure, monitor, fitted with different properly sized cuffs, was used for readings. Three consecutive readings, taken at one-minute intervals, and the two closest values were averaged to determine participant’s actual blood pressure used in the present study.

The authors reported that they relied on secondary data for this study. How did they train people to measure participant’s blood pressure? It is quite confusing

--- Thanks for bringing our attention to this issue. The study used primary data collected during the 2021 national survey. The training of the observers was carried out according to the international guidelines for the blood pressure measurement – this has been added to the text. We also added the following text (see page 5 lines 113 - 117):

*“All personnel who participated in administering the survey were trained health workers, mainly nurses from the Mauritian Ministry of Health and Wellness or government hospitals. The week before the survey, a workshop was held where every participant was specifically trained, first theoretically and then practically, under the supervision of the principal investigators. Each morning, all devices were tested, and those showing outlying values were not used.”*

Independent variables
Line 123: categorization of age was reported as 19-<45 years, 45– <55 years, 55-<65 years, 65-<75 years. Why not write as 19 – 45, 45 – 55 and 55 – 65 years. The question is, if someone is exactly 45 years which category will be person be placed 19-45 category or 45 – 55 category? This applies to the remaining categorizations. It needs to be done well.

--- The categories were named that way in order to clearly define de borders between them. For example, in categories such as 19 -<45 years, 45– <55 years, a person with exactly 45 years old are included in the category 45– <55 years as that person is in the lower level of that category while a person with less than 45 and 19 years or more will be included in the category 19 -<45 years. However, in order to simplify their names, they were renamed as follow: 19-44; 45-54; 55-64; 65-74; 75+

Additionally, is there any justification for wide variations in the categories comparing the first category (19-45 yrs) to the others.

---Given that the prevalence of hypertension was low in the population under 45, we decided to create the category 19-<45 years and use it as a reference for comparison with other age groups. This categorization allows us to more effectively analyze and highlight differences in hypertension prevalence across various age groups. By using the 19-44 years age group as a baseline, it is possible to better understand how the risk of hypertension increases with age.

Line 128: It is not allowed to combine “no formal education and elementary” as primary education. One who had “no formal education should be treated as had “no formal education”.

--- We agree that both groups could be different, however we decided to merge those two categories because there were very few individuals with no formal education (96 individuals had no formal education as opposed to 1094 with primary education). The category was renamed as: “primary or no formal education” to make clear that both education levels were included in one category.

Statistical analysis

The abstract is indicating 3622 as participants and the statistical analysis section is indicating 3607. Could you speak to the differences in the sample?

--- The abstract reports the total number of survey participants, which is 3622. The statistical analysis section, however, includes only 3607 participants. This is because 15 cases had missing values for the variable "hypertension" and were excluded from the analysis. Therefore, the analysis was conducted using only the 3607 cases with complete data for this variable.

Ethical considerations
The authors should indicate clearly if they were involved in the data collection exercise of the NCD survey. If yes, they should write on the detailed process of the informed consent process in addition to the ethics. If not, they should state that they were not involved in the data collection exercise and only relied on secondary data from the NCD survey. Then, they can state where (IRB) the NCD survey had ethical approval from and whether the study adhered to the declaration of Helsinki.

--- Thanks for the comment. Some of the authors were responsible for the planning and implementation of the current study. This has now been clarified in the text, together with the adherence to the Helsinki declaration. It is written in the Ethics section that the study received ethical approval from the National Ethical Committee in Mauritius and in Sweden.

Authors who participated directly in the survey:

- Jaysing Heecharan and Sudhirsen Kowlessur were always present.
- Bhushan Ori was present at start of the survey activities
- Stefan Söderberg and Jaakko Tuomilehto were present at workshop and 1st week of the data collection.

--- We have now added a sentence related to the Helsinki declaration (lines 193 - 195). It reads:

*“The study complied with the Helsinki declaration and the study reported in line with the Strengthening the Reporting of Observational Studies in Epidemiology (STROBE) guideline.”*

Results

Line 190: There is no very poor in Table 1 so I find it difficult to get where the very poor is from.

--- Thank you. We replaced the term “very poor” by “poorest” according to name of the category in the tables and other parts of the document.

Lines 234 – 245: Can the authors justify what could contribute to the differences in the hypertension prevalence compared with other countries.

--- Thanks for the comment. We have now added the following text in the discussion (please, see page 13, lines 254-259):

*“**Hypertension can vary among African countries due to differences in genetic factors, dietary habits, healthcare access, socioeconomic status, and lifestyle choices. A recent study assessing health systems challenges to control NCDs in Mauritius revealed a suboptimal coverage of population-based interventions combatting main NCD risk factors (tobacco smoking, harmful alcohol use, unhealthy diet and physical inactivity)* [22]*, which could partly explain the lack of decrease of the hypertension prevalence over time.”*

Line 259: How does excess body weight contributes to hypertension. Kindly add to the discussion.

---Thank you for this observation. The following text has been added to the discussion (please, see page 14, lines 279-281):

*“Excess body weight may lead to hypertension through impaired structural and functional renal activity, increased cardiac output, arterial and aortic stiffening due to the activation of sympathetic activity and the renin-angiotensin-aldosterone system (RAAS)* [33]*”*

Line 265: Just indicating that literature supports that alcohol consumption is linked to hypertension is not enough. Kindly add how it contributes to hypertension

--- Thank you for this comment. A brief text explaining how alcohol contributes to hypertension has now been added (please, see page 15, lines 289-294):

*“Alcohol has been shown to increase the risk of hypertension by increasing sympathetic nervous system activity, leading to increased heart rate and vasoconstriction, it also diminishes baroreceptor sensitivity, which impairs blood pressure regulation. Additionally, alcohol stimulates the RAAS , promoting sodium and water retention, leading to increased blood volume and peripheral resistance* [38,39]*”*

Lines 270: It appears misleading to commence the statement with an intriguing finding. This posits that you had a preconceived results you wanted to see or report on.

--- The relationship between smoking and hypertension has been widely reported, so we expected to observe a similar association in our study. Interestingly, no significant association was observed, and this result is reported accordingly.

The absence of a significant association between smoking and hypertension in our study does not negate the overall evidence of this relationship. It may indicate that the particular sample or context of the study differs from other populations or that other confounding factors are at play. It was explained in the discussion.

Conclusion

Lines 295 – 296 have no relation with the conclusion, kindly scrap it. I suggest the authors strengthen their concluding statement and prevent repetition of results.

--- The sentence “This study contributes to the growing body of literature on hypertension in Mauritius and provides valuable information for policymakers” has been removed from the conclusion. New text has been added to further strengthen that section. (please, see page 16, lines 327-335):

*“This study highlights the need for targeted interventions to address the growing prevalence of hypertension in Mauritius. Concerted efforts by the Mauritian Government should be made to promote health literacy and sustainable strategies that address modifiable risk factors for hypertension across all ages. There is also need for multisectoral collaboration to promote to risk factor awareness and emphasize primary and secondary prevention, especially among at-risk groups such as the elderly and Creole ethnicity. Additionally, further research is needed into the socioeconomic factors associated with hypertension and the barriers to the adherence with healthcare use for screening and treatment in this country with cost-free health services.”*

Implications for practice and research is completely missing.

---Thank you for this observation. It has now been included as part of the conclusion (please, see page 16, lines 327-335):

*“This study highlights the need for targeted interventions to address the growing prevalence of hypertension in Mauritius. Concerted efforts by the Mauritian Government should be made to promote health literacy and sustainable strategies that address modifiable risk factors for hypertension across all ages. There is also need for multisectoral collaboration to promote to risk factor awareness and emphasize primary and secondary prevention, especially among at-risk groups such as the elderly and Creole ethnicity. Additionally, further research is needed into the socioeconomic factors associated with hypertension and the barriers to the adherence with healthcare use for screening and treatment in this country with cost-free health services.”*

Reviewer #2: This paper reports data from a health survey in Mauritius. The data that are reported include prevalence of hypertension and factors associated with hypertension, limited by the survey questions and respondent self-reporting.

This report provides valuable insights into hypertension in Mauritius and further information on data from Sub-Saharan Africa.

Comments:
1. In general, the paper is well written. Minor language editing is required: the study aimed "to" (rather than "at"), "A cross-sectional national representative data" - presumably database?

--- “Aimed at” was replaced by “aimed to” as suggested.

--- Regarding the second suggestion, we refer to the representativeness of the collected data. We prefer to keep the original sentence.

2. The first sentence in the abstract may not be needed.

--- We acknowledge that the first sentence in the abstract may not be essential to the core findings of the study, but it provides a good introduction to the topic. So, we would prefer to keep it, but can drop it if the Editor will ask us to remove it.

3. Study setting might be better placed in the introduction.

--- Thanks for the suggestion but we have now modified the structured of the Methods section

4. You comment that lower education is associated with hypertension. However, the prevalence is lower in the lower education group (12.8% vs 32% and 48%) and the prevalence ratio is higher with higher education. Please clarify and discuss this further.

--- Thank you for your observation. However, our data actually show the opposite. The order of categories ranges from the highest (Tertiary) to the lowest level of education (Primary or no formal education). The prevalence of hypertension is 12.8% among individuals with the highest education level (Tertiary), whereas the highest prevalence (48.8%) of hypertension is found in the least educated group (Primary or no formal education).

Reviewer #3: I would like to suggest that the authors mention the approval number provided by Ethical Board Review Board of the country to conduct the survey. This study is not showing extraordinary report and delivers what is already known to the world. Hence, the co-relation of the stratified age group with smoking cigarettes, physical activity and hypertension would add quality to the paper.

--- The approval number provided by Ethical Board Review Board of the country to conduct the survey was added to the text (please, see page 8, line 194).

--- Thank you for the comment. We agree that the results are in line with many other studies looking at risk factors for hypertension. Its uniqueness however comes from the lack of updated data in this area nationally and the public health relevance of the findings for the Ministry of Health and Wellness of Mauritius.

--- We don’t think that adding more analysis would necessarily increase the quality of the paper. We interpret that the reviewer suggests some type of interaction analysis. Interaction analysis and stratification is needed in the investigation of effect modification – smoking and physical activity are risk factors that can be considered as risk factors or confounders, not effect modifiers. While possible to do, it would require some theoretical background of why to do it, a different research question and of course a different analytical approach. We believe this would be outside the scope of this specific manuscript.
