# Supplementary material for: Prevalence and risk factors of hypertension in Mauritius: A cross-sectional study
Source: PLOS Glob Public Health. 2024 Dec 12;4(12):e0003495. doi: 10.1371/journal.pgph.0003495 (PMC11637371; doi:10.1371/journal.pgph.0003495)
Supplement: Attachment — Submitted filename: review.docx [file pgph.0003495.s002.docx]

Thanks for the opportunity to rereview the manuscript. There is still a confusing in the manuscript; at some points authors report they use secondary data, at some points they report on data collection as if they actually collected primary data. The authors should clarify this by reporting as a study that utilised solely secondary data.

Abstract

The survey included 3,622 participants aged 19–84 years from all nine districts of the country. Please, report the sample size you used for the analysis in the abstract.

Materials and methods

Study settings

Could you add some information to the study setting?

Study design

We performed a cross-sectional study using the nationally representative data from 2021 100 Mauritius NCDs survey where 3,622 respondents (response rate 84.1%). I think your confusing stems from the fact that some of the authors participated in the survey. Once you stated that you used secondary data, it remains secondary data. As such, report on the data you used for the analysis and state why you excluded some of the participants where necessary.

Independent variables

Sex was divided into men and women. Revise to “male and female”

Results

Table 1

In the previous comment, I talked about how the table 1 should be presented, we keep going back. It is appropriate to have the % on the header section so you do not repeat in the entire table.

For example.

Variable	Frequency (n=3,607)	Percentage (%).

I do not know how you computed the prevalence. But, I had a different prevalence. If 1186 participants had hypertension out of 3,607, the overall prevalence cannot be 35.2%, it should be 32.9%.

Lines 230 – 239, please check and write the interpretation of the prevalence ratio well. I do not think you need to add % to the prevalence ratio when interpreting.

Study limitations

The study employed a standardized and comprehensive methodology to capture exposure and outcome variables including the careful and standardized measurement of blood pressure. Did you collect the data? You have reported that the study relied on secondary data, kindly report as such.
