# Supplementary material for: Prevalence and risk factors of hypertension in Mauritius: A cross-sectional study
Source: PLOS Glob Public Health. 2024 Dec 12;4(12):e0003495. doi: 10.1371/journal.pgph.0003495 (PMC11637371; doi:10.1371/journal.pgph.0003495)
Supplement: Attachment — Submitted filename: Response to reviewers 2024.10.10.docx [file pgph.0003495.s003.docx]

Dear Editor,

Thank you for your time in guiding the review process. We truly appreciate the insightful feedback from the reviewers, which has helped improve the quality of the manuscript.

All changes in the manuscript have been included using track changes. Responses to the reviewers' comments are provided in blue below each comment.

Kind regards,

Osvaldo Fonseca Rodríguez, on behalf of the authors

Thanks for the opportunity to rereview the manuscript. There is still a confusing in the manuscript; at some points authors report they use secondary data, at some points they report on data collection as if they actually collected primary data. The authors should clarify this by reporting as a study that utilised solely secondary data.

--- Thanks for the comment. We believe that this context, the data would still be considered primary data for the research group as a whole, even if some members of the group were not directly involved in data collection. Since the data were collected firsthand by members of the research group (even if not the first author), it formally qualifies as primary data. But we leave this conceptual issue to the consideration of the editor.

Abstract

The survey included 3,622 participants aged 19–84 years from all nine districts of the country. Please, report the sample size you used for the analysis in the abstract.

--- The number of respondents is 3622, the response rate was 84.1%, so the total contacted individuals was 4307. We added this information in the abstract of the current revised version.

Materials and methods

Study settings

Could you add some information to the study setting?

--- We have already provided information regarding the location of the country, its economy and population. We would appreciate if the reviewer could be more specific about the type of information that would like to read in this section.

Study design

We performed a cross-sectional study using the nationally representative data from 2021 100 Mauritius NCDs survey where 3,622 respondents (response rate 84.1%). I think your confusing stems from the fact that some of the authors participated in the survey. Once you stated that you used secondary data, it remains secondary data. As such, report on the data you used for the analysis and state why you excluded some of the participants where necessary.

--- In the last “response to reviewers” document we clarified this aspect. We are grateful to the reviewer for the opportunity to make it clear. It can be read as follow:

------------------------------------------------------------------------------------------------------------

Text from previous “Response to reviewer’s” document:

**The authors reported that they relied on secondary data for this study. How did they train people to measure participant’s blood pressure? It is quite confusing**

**--- Thanks for bringing our attention to this issue. The study used primary data collected during the 2021 national survey. The training of the observers was carried out according to the international guidelines for the blood pressure measurement – this has been added to the text. We also added the following text (see page 5 lines 113 - 117):**

***“All personnel who participated in administering the survey were trained health workers, mainly nurses from the Mauritian Ministry of Health and Wellness or government hospitals. The week before the survey, a workshop was held where every participant was specifically trained, first theoretically and then practically, under the supervision of the principal investigators. Each morning, all devices were tested, and those showing outlying values were not used.”***

------------------------------------------------------------------------------------------------------------

Independent variables

Sex was divided into men and women. Revise to “male and female”

--- Terms men and women were replaced with male and female across the manuscript.

Results

Table 1

In the previous comment, I talked about how the table 1 should be presented, we keep going back. It is appropriate to have the % on the header section so you do not repeat in the entire table.

For example.

Variable	Frequency (n=3,607)	Percentage (%).

--- The format of the table was changed according to the reviewer’s suggestion.

I do not know how you computed the prevalence. But, I had a different prevalence. If 1186 participants had hypertension out of 3,607, the overall prevalence cannot be 35.2%, it should be 32.9%.

--- Thank you very much for this comment. It gave us the opportunity to refine the descriptive analyses. The description of our sampled individuals was done before excluding those with missing data, which caused the mismatch in hypertension prevalence and other data between Table 1 and the text, as well as between Tables 1 and 2. All numbers are now correct.

As a result, Table 1 has been replaced, and the percentages in the text have been corrected.

Lines 230 – 239, please check and write the interpretation of the prevalence ratio well. I do not think you need to add % to the prevalence ratio when interpreting.

--- The prevalence ratio (PR) compares the prevalence of a condition between two groups. It is valid to interpret the PR in relative terms using percentage, e.g., a PR of 1.3 means a 30% higher prevalence compared to the reference group. However, to avoid misunderstandings with the interpretation, we have included the PR within parentheses, followed by its 95% CI in every case.

Study limitations

The study employed a standardized and comprehensive methodology to capture exposure and outcome variables including the careful and standardized measurement of blood pressure. Did you collect the data? You have reported that the study relied on secondary data, kindly report as such.

--- We collected the data as part of the 2021 national survey in Mauritius. This is primary data, and it has been mentioned in the manuscript after the first round of revisions. Please refer to the answer above regarding the “Study design”.
